# Microsomal triglyceride transfer protein -164 T > C gene polymorphism and risk of cardiovascular disease: results from the EPIC-Potsdam case-cohort study

**DOI:** 10.1186/1471-2350-14-19

**Published:** 2013-01-29

**Authors:** Romina di Giuseppe, Sonali Pechlivanis, Eva Fisher, Maria Arregui, Beate Weikert, Sven Knüppel, Brian Buijsse, Andreas Fritsche, Stefan N Willich, Hans-Georg Joost, Heiner Boeing, Susanne Moebus, Cornelia Weikert

**Affiliations:** 1 Department of Epidemiology, German Institute of Human Nutrition Potsdam-Rehbrücke, Nuthetal, Germany; 2 Department of Pharmacology, German Institute of Human Nutrition Potsdam-Rehbrücke, Nuthetal, Germany; 3 Institute for Medical Informatics, Biometry and Epidemiology, University Hospital of Essen, University Duisburg-Essen, Essen, Germany; 4 Administrative Office of the Commission on Genetic Testing, Robert Koch-Institute, Berlin, Germany; 5 Agency for Quality in Medicine, Berlin, Germany; 6 Department of Internal Medicine, Division of Endocrinology, Diabetology, Nephrology, Vascular Disease and Clinical Chemistry, University of Tübingen, Tübingen, Germany; 7 Institute for Social Medicine, Epidemiology and Health Economics and Chairman, Charité Center 1 for Humanities and Health Sciences, Charité University Medical Center, Berlin, Germany

**Keywords:** Epidemiology, Genetics, Myocardial infarction, Ischemic stroke, Cholesterol, Additive interaction

## Abstract

**Background:**

The microsomal triglyceride transfer protein (MTTP) is encoded by the *MTTP* gene that is regulated by cholesterol in humans. Previous studies investigating the effect of *MTTP* on ischemic heart disease have produced inconsistent results. Therefore, we have tested the hypothesis that the rare allele of the -164T > C polymorphism in *MTTP* alters the risk of cardiovascular disease (CVD), depending on the cholesterol levels.

**Methods:**

The -164T > C polymorphism was genotyped in a case-cohort study (193 incident myocardial infarction (MI) and 131 incident ischemic stroke (IS) cases and 1 978 non-cases) nested within the European Prospective Investigation into Cancer and Nutrition (EPIC)–Potsdam study, comprising 27 548 middle-aged subjects. The Heinz Nixdorf Recall study (30 CVD cases and 1 188 controls) was used to replicate our findings.

**Results:**

Genotype frequencies were not different between CVD and CVD free subjects (P = 0.79). We observed an interaction between the -164T > C polymorphism and total cholesterol levels in relation to future CVD. Corresponding stratified analyses showed a significant increased risk of CVD (HR_additve_ = 1.38, 95% CI: 1.07 to 1.78) for individuals with cholesterol levels <200 mg/dL in the EPIC-Potsdam study. HR_additive_ was 1.06, 95% CI: 0.33 to 3.40 for individuals in the Heinz Nixdorf Recall study. A borderline significant decrease in CVD risk was observed in subjects with cholesterol levels ≥200 mg/dL (HR_additve_ = 0.77, 95% CI: 0.58 to 1.03) in the EPIC-Potsdam study. A similar trend was observed in the independent cohort (HR_additve_ = 0.60, 95% CI: 0.29 to 1.25).

**Conclusions:**

Our study suggests an interaction between *MTTP* -164T > C functional polymorphism with total cholesterol levels. Thereby risk allele carriers with low cholesterol levels may be predisposed to an increased risk of developing CVD, which seems to be abolished among risk allele carriers with high cholesterol levels.

## Background

The microsomal triglyceride transfer protein (MTTP), a lipid transfer protein encoded by the *MTTP* gene, is located in the luminal side of the endoplasmic reticulum [1,2]. It plays an important role in the assembly and secretion of apolipoproteins B (ApoB) containing lipoproteins as chylomicrons in the intestine, and of very low density lipoproteins (VLDLs) in the liver [3-5]. In turn, chylomicrons transport exogenous lipids to cells, while VLDLs carry endogenous triglycerides through the bloodstream. Thus, considering the important role played by *MTTP* in fat absorption and lipoprotein regulation, several studies have been conducted on *MTTP* promoter polymorphisms [6-24].

Three polymorphisms of the *MTTP* gene (4q24), Ile128Thr (rs3816873), -164T > C (rs1800804), and -493G > T (rs1800591), have been described which are in complete linkage disequilibrium [6]. The rare alleles of these polymorphisms have been reported to decrease plasma lipoprotein-lipid levels [6-14] and some features of metabolic syndrome [16-19]. However, conflicting or negative findings have been reported by others [20-26]. The same inconsistent results have been published with regard to coronary heart disease (CHD) [7,24-26]. Two studies reported null findings [23,24]. In contrast, in the INTERGENE [26], ULSAM and WOSCOPS [7] studies homozygosity for the -164C and the -493T alleles was associated with increased risk of ischemic heart disease (IHD) though, in the latter, concomitantly to a decrease in total plasma cholesterol [7]. Nevertheless, considered the reduced expression of the *MTTP* gene in carriers of the rare alleles [26], it is reasonable to expect lower cholesterol levels which have previously been shown to regulate *MTTP* gene expression [27,28]. Also, it is likely to assume an interaction between the genotype of *MTTP* and cholesterol levels [19] in affecting the risk of CHD.

Therefore, in the framework of the European Prospective Investigation into Cancer and Nutrition (EPIC)–Potsdam study we investigated the association of the -164T > C variant, used as proxy for the three loci, with cardiovascular disease (CVD), myocardial infarction (MI) and ischemic stroke (IS) and the presence of an interaction between -164T > C polymorphism and total cholesterol in relation to CVD (MI and IS). We hypothesized that in carriers of the *MTTP* -164 C-allele lower cholesterol levels are associated with increased susceptibility to MI and IS.

## Methods

### Ethics statement

#### EPIC-Potsdam study

The Ethics Committee of the Medical Association of the State of Brandenburg approved the study procedures and a written informed consent was obtained from all participants.

#### Heinz Nixdorf Recall study

The study was approved by the local ethics committees, was conducted in accordance with German *Good Epidemiologic Practice* (GEP) including extended quality management procedures and re-certifications according to DIN ISO 9001:2001. Informed consent was obtained from all participants. Information on genotype, sex, age, diabetes, anti-hyperlipidemic drugs and CVD was available for up to 1 513 of 4 814 participants.

### Study population

#### EPIC-Potsdam study

Between 1994 and 1998, as a part of the large-scale European prospective cohort study EPIC, the EPIC-Potsdam study enrolled from the general population 16 644 women (aged 35 to 65 years) and 10 904 men (aged 40 to 65 years for), for a total of 27 548 participants [29].

At baseline, self-administered questionnaires on diet and lifestyle, PC-guided interviews with additional questions on prevalent diseases, blood pressure and anthropometric measurements were collected following standard procedures [30].

Every two years, information on incident diseases and changes in lifestyle are collected by self-administered questionnaires [31], with response rates during follow-up exceeding 90% at all occasions.

A case-cohort study consisting of all incident cases identified during a mean follow-up of 8 ± 2.2 years [32] and a subcohort of 2 500 individuals randomly selected from the EPIC-Potsdam cohort [33], was used to assess the association of the -164 T > C variant with CVD risk (including both MI and IS).

With this type of study, the results are expected to be representative of the entire cohort [34,35]. After the exclusion of participants with prevalent MI and stroke at the baseline, 269 individuals with incident MI and 246 individuals with incident stroke were identified (199 IS, 41 hemorrhagic strokes, and 6 strokes with undefined pathogenesis). Among the subcohort, non-ischemic strokes were not considered as cases, while for individuals who experienced both MI and IS only the first event was considered [36]. After exclusion of prevalent CVD and missing follow-up dates, 2 368 participants remained to be in the subcohort. After further exclusion of subjects with a history of diabetes at the baseline and/or treated with anti-hyperlipidemic drugs, and those with missing *MTTP* genotype, biochemical or covariates data the final case-cohort consisted of 2 302 individuals (324 CVD cases: 193 MI and 131 IS, and 1 978 non-cases). Among CVD cases, 37 were part of the subcohort.

#### Heinz Nixdorf Recall study

For the replication we analyzed data from the Heinz Nixdorf Recall (Risk Factors, Evaluation of Coronary Calcium and Lifestyle) study comprising 4 814 participants aged 45–75 years. The participants were randomly selected from registration lists of the densely populated Ruhr metropolitan area in Germany between 2000 and 2003. The rationale and design of the study have been described in detail [37]. Between 2006 and 2008 incident cases were identified during the 5-year follow-up examination.

The genotyping of the *MTTP* SNP –I128T (rs3816873) was already available in a random selected sample of n = 1 513 Heinz Nixdorf Recall participants out of 4 814. After exclusion of participants with a history of CVD and/or diabetes at baseline and/or treated with anti-hyperlipidemic drugs, the final replication cohorts consisted of n = 1 218 individuals (30 CVD and 1 188 non-cases).

### Ascertainment of CVD

#### EPIC-Potsdam study

As described elsewhere [32], all possible cases of MI or stroke were identified by self-report or by death certificate in one of the four follow-up questionnaires and further verified by contacting the patients’ attending physician or by review of death certificates according to WHO MONICA criteria.

#### Heinz Nixdorf Recall study

Incident CVD (n = 30) included fatal and nonfatal MI (n = 24) and other CVD (n = 6), were identified.

Primary endpoints for this study were based on unequivocally documented incident coronary events that met predefined study criteria. We considered a myocardial infarction event based on symptoms, signs of electrocardiography, and enzymes (levels of creatine kinase (CK-MB)) as well as troponin T or I, and necropsy as 1) non-fatal acute myocardial infarction and 2) coronary death, which occurred between the baseline examination and five years after study entry [38,39]. For all primary study endpoints, hospital and nursing home records including electrocardiograms, laboratory values, and pathology reports were collected. For deceased subjects, death certificates were collected and interviews with general practitioners, relatives and eyewitnesses were undertaken if possible. Medical records were obtained in 100% of all reported endpoints. An external criteria and endpoint committee blinded for conventional risk factor status and CAC scores reviewed all documents and classified the endpoints thereafter. Due to the small number of cases only total CVD were considered for the replication analysis.

### Other measurements

Prevalent diabetes mellitus was identified by a physician and based on self-reported medical diagnoses, medication records and dieting behavior. Prevalent hypertension was defined as follows: systolic blood pressure ≥140 mm Hg or diastolic blood pressure ≥90 mm Hg or self-reporting of a diagnosis or use of antihypertensive medication. Education, lifestyle characteristics (including alcohol consumption), regular sport activity and smoking history were assessed at baseline by trained interviewers during a PC-guided interview. Trained personnel took anthropometric and blood pressure measurements.

### Biochemical analyses

#### EPIC-Potsdam study

At baseline, 30 ml of venous blood was taken from the respected participant (non-fasting or fasting blood) and, after fractionation into serum, plasma (collected on citrate, 10% of total volume), leukocytes, and erythrocytes immediately stored at -196° in liquid nitrogen [31]. All biomarkers were determined in 2007 in the Department of Internal Medicine, University of Tübingen. Plasma glucose, high-density lipoprotein cholesterol (HDL), total cholesterol and triglycerides were measured with an automatic analyzer (ADVIA 1650, Siemens Medical Solutions, Erlangen, Germany). LDL-cholesterol was calculated using Friedewald’s formula [40]. To account for citrate’s dilution factor concentrations of total, HDL-, LDL-cholesterol and triglycerides were multiplied by 1.1.

#### Heinz Nixdorf Recall study

At baseline, plasma cholesterol levels were measured with an automatic analyzer (ADVIA 1650, Siemens Medical Solutions, Erlangen, Germany).

### Genetic analyses

#### EPIC-Potsdam study

DNA extraction was performed using a commercial kit (Qiagen, Hilden, Germany). In 2009 at the Max Delbrück Center for Molecular Medicine, Berlin, Germany, the *MTTP* SNP -164T > C (rs1800804) was genotyped by TaqMan technology (Applied Biosystems, Foster City, CA, USA) using 5 ng of whole-genome amplified DNA per sample. The call rate for the SNP assay exceeded 98%.

#### Heinz Nixdorf Recall study

Lymphocyte DNA was isolated from EDTA anti-coagulated venous blood by a Chemagic Magnetic Separation Module I (Chemagen, Baesweiler, Germany). The *MTTP* SNP –I128T (rs3816873) was genotyped using four different platforms: Illumina Hap300, Illumina Hap550, Illumina Human660W-Quad and Illumina HumanOmni1-Quad. The call rate for this SNP was 99.9%.

### Statistical analysis

For both EPIC-Potsdam and Heinz Nixdorf Recall studies statistical analyses were performed with the use of SAS software package, release 9.2 (SAS Institute, Cary, NC).

#### EPIC-Potsdam study

Data on triglyceride measurements were transformed into natural logarithms to reduce skewness and data were reported as geometric means and 95% confidence interval (CI).

The deviation from Hardy-Weinberg equilibrium (HWE) was measured using the χ^2^ test.

The HWE was tested in the subcohort. Age and sex adjusted analysis of variance was used to describe general characteristics according to -164T > C genotype. Data were reported as means and standard error (SE). P for trend was calculated from age and sex adjusted linear regression model. To investigate the impact of total cholesterol on the associations between genotype and CVD we performed stratified analyses according to low (<200 mg/dL) and borderline-high/high (≥200 mg/dL) cholesterol levels as defined in the Adult Treatment Panel III (ATP III) report [41]. Multiplicative interaction between cholesterol levels and genotype as well as sex and genotype in relation to CVD was tested with cross-product term. As a measure of additive interaction between cholesterol levels and genotype we further calculated the Synergy Index (SI) and the relative excess risk due to interaction (RERI) and their 95% confidence interval (CI) [42], as suggested by Rothman [43]. SI > 1 and RERI > 0 suggest a positive interaction (superadditive effect); SI < 1 and RERI < 0 suggest a negative interaction (subadditive effect); SI = 1 and RERI = 0 suggest absence of interaction.

Both the multiplicative and additive interactions were also tested for triglycerides, HDL- and LDL-cholesterol. The choice of the cut-off values for these biomarkers was also based on the ATP III criteria (lower and higher than 130 mg/dL for LDL-cholesterol; lower and higher than 40 mg/dL and lower and higher than 50 mg/dL for HDL-cholesterol, respectively, in men and women; lower and higher than 150 mg/dL for triglycerides) [41].

Cox proportional-hazard regression modified according to the Prentice method [34] was used to compute the age and sex adjusted hazard ratio (HR) and 95% CI for the associations between *MTTP* -164T > C and risk of MI, IS and total CVD (combined endpoints). In the counting processes age was the underlying time variable with “entry time” defined as age at baseline and “exit time” as age at CVD event (MI or IS) or censoring. Associations between *MTTP* -164T > C and CVDs were tested in three models: additive, dominant and recessive. Furthermore, competing risk analyses were performed to test whether the associations of *MTTP* -164T > C with cardiovascular events differed between MI and IS, as described by Lunn and McNeil [44].

The power to detect an association between the *MTTP* -164T > C SNP and CVDs (MI, IS and combined endpoints) was computed with Quanto (http://hydra.usc.edu/GxE/) [45], in relation to a desirable power = 80%, assuming α = 0.05 and a disease prevalence of 3.1% for CVD (n = 847), 2.0% for MI (n = 544) and 1.1% for IS (n = 303), reflecting the baseline prevalence data in the EPIC-Potsdam population. The detectable odds ratio per risk allele equals 1.30, 1.39 and 1.47, respectively for CVD, MI and IS.

#### Heinz Nixdorf Recall study

Cox proportional-hazard regression was used to compute the age and sex adjusted hazard ratio (HR) and 95% CI for the associations between *MTTP* –I128T and risk of total CVD. Associations between *MTTP* –I128T and CVDs were tested using the additive model.

## Results

### General characteristics

The genotype distribution of the -164T > C and –I128T SNPs followed the HWE (*P* = 0.37 and *P* = 0.71, respectively for the EPIC-Potsdam subcohort and for the replication Heinz Nixdorf Recall study). There were 1 114, 758 and 143 subjects with genotypes TT, CT, CC respectively (738 men and 1 277 women) observed in the subcohort. The C allele frequency was 0.26 for both studies. According to -164T > C genotype, age- and sex-adjusted baseline characteristics of subjects who did or did not develop cardiovascular events during the follow-up period are shown in Table 1. In particular, subjects with incident CVD (n = 324; 193 incident MI; 131 incident IS) were men, older, smokers and with a lower educational level than individuals who remained free of CVD (n = 1 978) during a mean follow-up of 8.2 years. Furthermore, they had a higher prevalence of abdominal obesity and hypertension, slightly higher total and low density lipoprotein- (LDL-) and lower HDL-cholesterol levels (Table 1). According to genotype no significant differences in central obesity, obesity and hypertension and socio-demographic characteristics were observed in subjects with or without CVD (Table 1). In contrast, opposite trends were observed for total-, LDL-cholesterol and triglyceride levels. In the group free of CVD carriers of the C-allele showed slightly higher total- and LDL-cholesterol levels (P_trend_ = 0.036 and P_trend_ = 0.026, respectively). In the group of CVD carriers of the C-allele showed lower triglyceride (P_trend_ = 0.016) along with slight lower total-cholesterol levels (P_trend_ = 0.033) (Table 1).

**Table 1:** **Baseline characteristics of subjects according to cardiovascular disease status and *MTTP *-164 T > C genotype in EPIC-Potsdam study**

		**CVD free by genotype**					**CVD by genotype**			
	**All CVD free**	**TT**	**CT**	**CC**	**P for trend**^**a**^	**All CVD**	**TT**	**CT**	**CC**	**P for trend**^**a**^
n	1 978	1 096	742	140		324	178	126	20	
age, yrs	49.8 ± 0.2	49.5 ± 0.3	49.2 ± 0.3	49.4 ± 0.7	0.543	54.9 ± 0.5	56.1 ± 0.6	54.1 ± 0.7	56.6 ± 1.7	0.223
Men, %	36.8	36.7	35.3	35.0	0.687	58.5	64.3	60.9	69.4	0.654
Abdominal obesity, %^b^	21.4	20.9	20.3	18.8	0.567	26.9	32.2	30.9	30.0	0.837
Obesity, %^c^	14.9	14.5	15.5	9.7	0.134	18.4	19.9	19.4	19.6	0.975
Hypertension, %	47.6	45.6	44.7	43.7	0.592	60.3	70.8	68.3	49.0	0.114
Current smokers, %	21.4	21.2	22.5	21.4	0.957	39.7	35.7	31.6	47.9	0.264
High education, %	41.7	44.2	38.7	44.4	0.978	34.1	31.8	30.3	36.1	0.699
High sport activity, %	24.8	25.2	24.4	27.5	0.230	19.9	18.0	13.0	23.5	0.534
Cholesterol, mg/dL	191 ± 0.9	189 ± 1.2	192 ± 1.5	195 ± 3.4	0.036	198 ± 2.3	210 ± 3.3	199 ± 3.9	199 ± 9.7	0.033
HDL-cholesterol, mg/dL	52 ± 0.3	52 ± 0.4	52 ± 0.5	52 ± 1.2	0.840	50 ± 0.8	50 ± 1.1	51 ± 1.3	47 ± 3.2	0.575
LDL-cholesterol, mg/dL	114 ± 0.8	112 ± 1.0	115 ± 1.2	116 ± 2.8	0.026	120 ± 1.9	128 ± 2.7	121 ± 3.2	129 ± 7.9	0.305
Triglyceride, mg/dL^d^	107 (105-110)	105 (101-108)	108 (104-112)	114 (104-124)	0.087	118 (111-125)	131 (120-144)	116 (104-129)	99 (76-129)	0.016
Alcohol consumption, g/day	8.0 (7.5-8.5)	8.2 (7.6-8.9)	7.5 (6.8-8.2)	9.5 (7.6-11.7)	0.871	5.9 (5.1-6.9)	5.5 (4.3-7.0)	5.8 (4.4-7.6)	3.9 (2.0-7.9)	0.663

^a^Determined from linear regression model adjusted for age and sex (where appropriate) in reference to CVD free and CVD by genotype. Age was adjusted for sex. Sex was adjusted for age.

^b^Abdominal obesity was defined according to the ATP III criteria [41] based on the following waist circumference cut-off points: men ≥ 102 cm and women ≥ 88 cm.

^c^Obesity was defined as BMI ≥ 30 kg/m^2^.

^d^Geometric means and 95% (CI) all such values.

### Association between ***MTTP***-164T > C polymorphism and incident CVD

The association between the *MTTP* -164T > C polymorphism and CVD events was tested also for MI and IS separately, taking into consideration the additive, dominant and recessive models (Table 2). Since there were no sex differences in the association between *MTTP* -164T > C and CVD (P for interaction = 0.86), we combined men and women in all analyses. After adjustment for age and sex, Cox regression analyses revealed no significant association between the -164T > C variant and CVD risk considering the additive (HR_additive_ = 1.04, 95% CI: 0.86 to 1.25; P = 0.714), dominant (HR_CT+CC vs TT _= 1.09, 95% CI: 0.85 to 1.39; P = 0.505) and recessive (HR_CC vs CT+TT_ = 0.90, 95% CI: 0.55 to 1.46; P = 0.662) models. Nevertheless, the multiplicative and additive interactions between the -164T > C polymorphism and total cholesterol (dichotomous) in relation to CVD risk were significant and in the same negative direction (β_multiplicative interaction_ = -0.55 ± 0.19; P = 0.004; SI_additive interaction_ = 0.31, 95% CI: 0.16 to 0.62 and RERI_additive interaction_ = -1.44, 95% CI: -2.37 to -0.51).

**Table 2:** **Hazard rate ratios (HR) and 95% confidence intervals (95% CI) for the associations between *MTTP *-164 T/C polymorphism, CVD (combined endpoints), MI and IS**

	**Cholesterol < 200 mg/dL**			**Cholesterol ≥ 200 mg/dL**		
	**HR (95% CI)**^**a**^			**HR (95% CI)**^**a**^		
	CVD	MI	IS	CVD	MI	IS
Cases, n	139	69	70	185	77	40
C allele	1.38 (1.07-1.78)	1.19 (0.82-1.72)	1.60 (1.16-2.20)	0.77 (0.58-1.03)	0.76 (0.54-1.07)	0.77 (0.48-1.23)
P_additive_	0.014	0.353	0.004	0.075	0.113	0.273
Dominant	1.76 (1.22-2.54)	1.41 (0.85-2.33)	2.22 (1.35-3.64)	0.67 (0.48-0.94)	0.65 (0.43-0.97)	0.69 (0.40-1.18)
P_dominant_	0.002	0.186	0.002	0.021	0.036	0.172
Recessive	0.73 (0.31-1.69)	0.71 (0.21-2.39)	0.75 (0.25-2.27)	0.98 (0.53-1.82)	0.99 (0.47-2.06)	0.93 (0.33-2.66)
P_recessive_	0.458	0.580	0.616	0.948	0.969	0.892

^a^Adjusted for age and sex.

Stratified analyses according to low (<200 mg/dL) and borderline-high/high (≥200 mg/dL) cholesterol levels showed significant positive associations between *MTTP* -164T > C and CVD in subjects with cholesterol levels <200 mg/dL, considering both the additive (HR_additive_ = 1.38, 95% CI: 1.07 to 1.78; P = 0.014) and the dominant models (HR_CT+CC_ = 1.76, 95% CI: 1.22-2.54; P = 0.002) (Table 2). Analyzing MI and IS separately, the associations seemed to be stronger for stroke (HR_additive_ =1.60, 95% CI: 1.16 to 2.20, P = 0.004; HR_dominant_ = 2.22, 95% CI: 1.35 to 3.64, P = 0.002) than for MI (HR_additive_ = 1.19, 95% CI: 0.82 to 1.72, P = 0.353; HR_dominant_ = 1.41, 95% CI: 0.85 to 2.33, P = 0.186). However, results from the competing risk analysis (IS versus MI) provided a Wald test P value equal to 0.28 and 0.20, respectively, for the additive and dominant model. Further adjustment for other CVD risk factors (i.e. body mass index, waist circumference, prevalent hypertension, sport activity and alcohol consumption) led to similar HRs depicted in Table 2 (data not shown).

In subjects with cholesterol levels ≥200 mg/dL we observed a borderline inverse association between *MTTP* -164T > C and CVD in the additive model (HR_additive_ = 0.77, 95% CI: 0.58 to 1.03, P = 0.075) and significant relationships in the dominant model (HR_dominant_ = 0.67, 95% CI: 0.48 to 0.94, P = 0.021; HR_dominant_ = 0.65, 95% CI: 0.43 to 0.97, P = 0.036, respectively, for CVD and MI) (Table 2).

We performed additional analyses to test both the multiplicative and additive interactions for triglycerides, HDL- and LDL-cholesterol concentrations. The interactions between *MTTP*/triglycerides and *MTTP*/HDL-cholesterol in relation to CVD were not significant (P = 0.18 and P = 0.11, respectively), whereas they were significant and in the same direction as those found for total cholesterol when LDL-cholesterol was analyzed (multiplicative interaction: P = 0.023; SI_additive interaction_ = 0.33; 95% CI: 0.15 to 0.73 and RERI_additive interaction_ = -1.17; 95% CI: -2.01 to -0.33). Stratified analysis according to the 2 LDL-cholesterol categories (<130 and ≥130 mg/dL) showed an increased CVD (HR_additive_ = 1.24; 95% CI: 0.98 to 1.56; HR_dominant_ = 1.51; 95% CI: 1.09 to 2.08) and IS risk (HR_additive_ = 1.30; 95% CI: 0.96 to 1.75; HR_dominant_ = 1.66; 95% CI: 1.07 to 2.57) in the low LDL group when *MTTP* was considered in a dominant fashion. A decreased CVD (HR_additive_ = 0.80; 95% CI: 1.58 to 1.09; HR_dominant_ = 0.69; 95% CI: 0.47 to 1.00) and MI (HR_additive_ = 0.74; 95% CI: 0.51 to 1.07; HR_dominant_ = 0.62; 95% CI: 0.39 to 0.96) risk was observed, instead, in the high LDL group, always in a dominant fashion (data not shown).

In the replication cohort we did observe a trend toward a decreased CVD risk in individuals with cholesterol levels higher than 200 mg/dL (HR_additive_ = 0.60, 95% CI: 0.29 to 1.25; P = 0.17). No association was observed in the other strata (<200 mg/dL) (HR_additive_ = 1.06, 95% CI: 0.33 to 3.40; P = 0.92).

## Discussion

In this study, we anticipated an interaction between total cholesterol levels and the *MTTP* -164T > C polymorphism with regard to the CVD risk. The presence of a statistically significant interaction confirmed our hypothesis and indicated carriers of the C allele of the *MTTP* -164T > C polymorphism with plasma total cholesterol levels lower than 200 mg/dL had an increased risk of CVD. The association seemed to be stronger for IS than for MI, but differences in the associations were not supported by competing risk analysis. Conversely, the *MTTP* -164 C-allele showed a lower CVD, and MI, risk in participants with cholesterol levels higher than 200 mg/dL. Similar relationships were observed considering LDL-cholesterol with levels lower and higher than 130 mg/dl suggesting that LDL is the driving cholesterol component. However, the value of LDL levels seems to be limited as they had to be estimated based on the Friedewald formula [46]. In fact further studies are needed to replicate these findings.

The association between *MTTP* –I128T polymorphisms and CVD risk observed in the replication cohort showed a similar trend within the strata of cholesterol levels higher than 200 mg/dL. However, considering that the number of cases in the Heinz Nixdorf Recall study is small further replication studies are needed. To our knowledge, this is the first prospective study showing such an effect of *MTTP* on risk of IS.

With regard to the association between the *MTTP* -164T > C polymorphism and cholesterol levels, previous studies observed inconsistent results. Few studies reported a slight cholesterol lowering effect of the rare alleles of the *MTTP* promoter polymorphisms [7,10]. Ledmyr et al. investigated the association between the *MTTP* -493 G/T polymorphism and cholesterol in both healthy and hyper-cholesterolemic individuals, and observed decreased levels of total cholesterol in carriers of the -493 T variant [7,10]. Furthermore, Phillips et al. in a small study including 82 patients with type 2 diabetes mellitus (T2DM) of a Caucasian population found that the subjects heterozygous for the -493 G/T had lower LDL-cholesterol and, in the postprandial phase, higher apoB48 levels in the VLDL fraction. The authors suggested that the -493 G/T polymorphism seemed to confer protection against atherosclerosis in T2DM patients [12]. In contrast, Jou et al. observed that total cholesterol, LDL-, and non HDL-cholesterol levels were higher according to the rare allele of the MTTP -493 G/T polymorphism when disease free young African Americans were investigated [19,20]. Further, Lundahl et al. observed lower serum triglyceride levels in subjects affected by familial hypercholesterolemia and homozygous for the rare allele of the MTTP -493 G/T genotype [8].

Overall, these studies seem to suggest that *MTTP* regulates lipids differently in the presence or absence of disease, although the occurrence of a possible interaction between the LDL receptor and the *MTTP* genes is not excluded [7,14]. Our results seem to be in line with these hypotheses. On one hand we observed slightly higher total and LDL-cholesterol levels in subjects free of CVD and homozygous for the rare -164 C allele, and on the other, lower total cholesterol and triglyceride levels according to the rare allele of the *MTTP* -164T > C polymorphism in the group of future CVD cases.

It has been shown that the C–allele of the *MTTP*-164 T > C polymorphism is homologous to a putative sterol response element (SRE) binding site and as such confers a reduced *MTTP* expression [26-28]. These findings come from an experimental study in which Hagan et al. demonstrated that human *MTTP* promoter activity is up-regulated by cholesterol [27]. The mechanism based on which cholesterol regulates *MTTP* gene expression is linked to the presence of a modified SRE in the *MTTP* promoter [27]. When cholesterol levels are low, the sterol regulatory element binding protein (SREBP) acts as transcription factor, binds to the SRE thereby inhibiting *MTTP* gene expression [28]. In contrast, in presence of cholesterol the modified SRE likely binds a new SREBP family member thus up-regulating *MTTP* expression [27,28]. These observations suggest that *MTTP* gene expression is differently regulated by high and low cholesterol levels.

Despite the lack of significant associations between the -493G > T or -164T > C single nucleotide polymorphisms (SNPs), coronary heart disease and blood lipids observed in two previous studies [24,25], recently Aminoff et al. put forward that carriers of the rare -164C allele are at increased risk of IHD [26]. They substantiated their findings by showing *in vivo* that the presence of the rare alleles of the -493G > T and -164T > C SNPs confer lower *MTTP* transcription in the heart, liver and macrophage. This mechanism, in turn, by causing the lipid accumulation in the heart would provoke an increased IHD risk. Indeed, our findings are in line with those of Aminoff et al. though they concluded that the increased IHD risk observed according to the -164C variant was independent of plasma lipids. As mentioned above, because human *MTTP* promoter activity is positively regulated by cholesterol [27], it is reasonable to assume that subjects with low cholesterol levels have, in general, a lower *MTTP* gene expression. Thus, in this low risk group carriers of the -164C variant, compared to carriers of the common allele, might be at increased CVD because of their lower *MTTP* gene expression. At the same time, if one would consider the observed associations as those mimicking *MTTP* inhibitors, then these findings could further highlight the concerns expressed by Aminoff et al. regarding the long term side effects *MTTP* inhibition may generate [26,47].

In contrast, in subjects with higher cholesterol levels we observed a reduced, though borderline significant, CVD risk accordingly to the *MTTP* gene -164 C variant. Our findings suggest that there could be an antagonistic (qualitative) interaction between cholesterol levels and *MTTP* -164 T > C polymorphism. These observations warrant further investigation.

The main limitation of this study is that the plasma lipoprotein and apolipoprotein levels, which are important in the effect of *MTTP* in cardiovascular disease, were not measured; our analyses on triglyceride levels were based on both fasting and non-fasting subjects; we estimated the LDL-cholesterol levels based on Friedewald equation. Strength of our study includes its prospective design. Furthermore, all cases of MI and IS were validated by medical records and were derived from a cohort population with a very high follow-up coverage.

## Conclusions

The findings of this study suggest that in the subjects investigated an interaction between *MTTP* -164T > C functional polymorphism with total cholesterol levels predisposes risk allele carriers with low cholesterol levels to an increased risk of developing CVD, which seems to be abolished among risk allele carriers with high cholesterol levels. However, further studies are warranted in order to shed more light on these complex mechanisms.

## Competing interests

The authors declare that they have no competing interest.

## Authors’ contributions

Conceived and designed the research: HGJ CW HB. Acquired the data: EF AM CW HB. Analyzed the data: RdG SP. Interpreted the data: RdG EF BW SK SNW HB CW. Wrote the manuscript: RdG. Critical revision of the article for important intellectual content: SP EF MA BW SK BB AF SNW HGJ HB SM CW. All authors read and approved the final manuscript.

## Pre-publication history

The pre-publication history for this paper can be accessed here:

http://www.biomedcentral.com/1471-2350/14/19/prepub
